# Identification of invasion-metastasis associated MiRNAs in gallbladder cancer by bioinformatics and experimental validation

**DOI:** 10.1186/s12967-022-03394-8

**Published:** 2022-04-28

**Authors:** Jiasheng Cao, Huijiang Shao, Jiahao Hu, Renan Jin, Anyun Feng, Bin Zhang, Shijie Li, Tianen Chen, Sarun Jeungpanich, Win Topatana, Yitong Tian, Ziyi Lu, Xiujun Cai, Mingyu Chen

**Affiliations:** 1 Department of General Surgery, Sir Run-Run Shaw Hospital, Zhejiang University, No. 3 East Qingchun Road, Hangzhou, 310016 Zhejiang Province China; 2 Zhejiang University School of Medicine, Zhejiang University, Hangzhou, 310058 Zhejiang Province China; 3 Department of Hepatobiliary and Pancreatic Surgery, Shaoxing People’s Hospital, Shaoxing, 312000 Zhejiang Province China; 4 Health Management Center, The First Affiliated Hospital, College of Medicine, Zhejiang University, Hangzhou, 310022 China

**Keywords:** Gallbladder cancer, MiRNA, Invasion, Metastasis, Bioinformatics

## Abstract

**Background:**

Recent studies exploring the roles of invasion-metastasis associated miRNAs in gallbladder cancer (GBC) are limited. In the study, we aimed to identify the invasion-metastasis associated miRNAs in GBC by bioinformatics and experimental validation.

**Methods:**

MiRNAs of different expression were identified by comparing GBC tumor samples with different survival from Gene Expression Omnibus database. MiRTarBase was used for identifying the potential target genes of miRNAs. Then, we performed Gene Ontology (GO) analysis and Kyoto Encyclopedia of Genes and Genomes (KEGG) pathway enrichment analysis. And miRNA-gene and protein–protein interaction (PPI) network were constructed for hub genes evaluation. We further explored and compared miR-642a-3p and miR-145-5p expression in both The Cancer Genome Atlas database and our hospital data. Finally, quantitative real-time PCR, wound healing assay, and Transwell assay were conducted to validate the invasion-metastasis associated miRNAs in GBC.

**Results:**

In GSE104165 database, 25 up-regulated and 97 down-regulated miRNAs were detected with significantly different expression in GBC tumor samples. Then, 477 potential target genes were identified from the 2 most up-regulated miRNAs (miR-4430 and miR-642a-3p) and 268 genes from the 2 most down-regulated miRNAs (miR-451a and miR-145-5p). After GO and KEGG analysis, mTOR and PI3K-Akt signaling pathways were found associated with the potential target genes. Based on PPI network, the top 10 highest degree hub nodes were selected for hub genes. Furthermore, the miRNA-hub gene network showed significant miR-642a-3p up-regulation and miR-145-5p down-regulation in both GBC tissues and cell lines. In the experimental validation, miR-145-5p up-regulation and miR-642a-3p down-regulation were confirmed to suppress GBC invasion and metastasis.

**Conclusions:**

MiR-642a-3p and miR-145-5p were identified as invasion-metastasis associated miRNAs via bioinformatics and experimental validation, and both up-regulation of miR-642a-3p and down-regulation of miR-145-5p would be served as novel treatment options for GBC in the future.

**Supplementary Information:**

The online version contains supplementary material available at 10.1186/s12967-022-03394-8.

## Background

Gallbladder cancer (GBC) is the most common type of biliary tract cancer (BTC), which has a high mortality and a poor prognosis [1–4]. According to the 8th American Joint Committee on Cancer (AJCC) guideline [5], surgical resection is the best potential treatment for GBC at an early stage, while chemotherapy/radiotherapy/immunotherapy/targeted therapy is recommended for GBC at an advanced stage. Patients with advanced GBC have limited response to treatment and poor prognosis due to the highly invasive and metastatic characteristics of GBC, including local tumor growth, hepatic invasion, and lymph nodes metastasis [6, 7]. Several studies reported the crucial role of multiple biological processes in cancer invasion and metastasis, such as circulating cancer cells, immune evasion [8], epithelial-mesenchymal transition (EMT) [9, 10], and cancer stem cells [11], while the multi-step process and the molecular mechanism of GBC remained unclear. Therefore, it is urgent to explore the novel biomarkers associated with invasion and metastasis in GBC patients to improve the prognosis.

MiRNAs, the small single-stranded non-coding RNAs (ranging from 18 to 25 bp in length), are critical in tumor development, such as invasion, migration, and metastasis [12, 13]. Different expression of miRNAs has been detected in various cancers. For example, miR-15a/16-1 could attenuate immunosuppression and represent a potential immunotherapy against hepatocellular carcinoma [14], meanwhile, miR-124-3p could promote cell proliferation in intrahepatic cholangiocarcinoma [15]. Additionally, by modulating STAT3 signaling, miR-124-3p was identified to suppress programmed cell death-ligand 1 (PD-L1) expression and inhibit tumorigenesis of colorectal cancer [16]. Nevertheless, as a highly aggressive cancer, studies exploring the potential roles of invasion-metastasis associated miRNAs in GBC are limited.

In the study, we aimed to conduct bioinformatic analysis to identify the invasion-metastasis associated miRNAs in GBC, and experimental validation were further performed to confirm the role of miRNAs. In turn, the study would provide potential effective treatment for improving the prognosis of GBC patients.

## Methods

### Data collection

Datasets of miRNA expression of GBC tissues with survival data from the National Center for Biotechnology Information (NCBI) (https://www.ncbi.nlm.nih.gov/geo) online Gene Expression Omnibus (GEO) database were included in the study. The search strategy was “gallbladder cancer” [MeSH Terms] AND “miRNA” [MeSH Terms]. After screening the datasets, the GSE104165 dataset, which contained 40 human GBC tumor samples and 8 normal gallbladder samples with survival data, was selected for further analysis. The sample platform was GPL18402 Agilent-046064 Unrestricted_Human_miRNA_V19.0_Microarray.

### Screening miRNAs of different expression

The miRNA expression data profile in txt format were preprocessed. Limma package was applied to calculate the miRNA of different expression from the Bioconductor package (http://www.bioconductor.org/). MiRNAs of different expression were found by comparing GBC tumor samples with long survival and short survival in R Studio (Version 4.0.4). Different expression was determined as significant when *P*-value < 0.05 and |fold change (FC)|> 1.

### Target genes prediction and gene ontology (GO) analysis

MiRTarBase (http://mirtarbase.mbc.nctu.edu.tw/php/index.php), an online miRNA-target interactions database, was used for identifying the potential target genes of the up-regulated or down-regulated miRNAs. The data were further validated by microarray, western blot, reporter assay, and next-generation sequencing experiments. GO analysis was extensively used for the determination of unique biological attributes of genes, gene products, and sequences, such as molecular function, cell components, and biological process [17]. Enrichr (http://amp.pharm.mssm.edu/Enrichr/), a 2016 updated webserver with a comprehensive enrichment analysis of the gene set, was introduced to manipulate enrichment analysis of the predicted target genes pathway and functional annotation, which was further analyzed for GO and Kyoto Encyclopedia of Genes and Genomes (KEGG) pathways [18].

### MiRNA-gene and protein–protein interaction (PPI) network construction

Tools were used to determine the interactions between genes or proteins to establish the miRNA-gene and PPI network. The target genes were first entered to assess functional associations in the STRING database (http://string-db.org) [19]. Next, the connectivity degree of PPI network was calculated in Cytoscape software (Version 3.6.0) for further hub genes evaluation. Finally, a miRNA-hub gene regulatory network was generated.

### The cancer genome atlas (TCGA) database and survival data of GBC patients

The BTC miRNA-sequencing data and clinical information were retrieved from TCGA database (https://portal.gdc.cancer.gov) to explore miR-642a-3p and miR-145-5p expression levels in BTC tissues. The data of overall survival (OS) were matched with miR-642a-3p and miR-145-5p expression values. A total of 41 GBC specimens were randomly selected from curative resection GBC patients retrospectively in Sir Run-Run Shaw Hospital (SRRSH), Hangzhou, Zhejiang Province, China. The interval between surgery and death was defined as OS. The follow-up period was defined as the duration between surgery and recurrence or death. The final follow-up was in December 2019.

### Validation of potential target genes using TCGA database

Based on short and long survival of GBC patients in TCGA database, the related mRNA expression (*SYK*, *SH3GL1*, *CDKN1A*, *MYC*, *VEGFA*, and *EGFR*) were compared between two groups. Moreover, the expression of some potential mRNA (*CDKN1A*, *MYC*, *VEGFA*) was further compared based on different T stages, which represented tumor invasion.

### Cell culture and tissue collection

Human GBC cell line GBC-SD and human intrahepatic biliary epithelial cells (HiBEC) were purchased from a cell bank (Chinese Academy of Sciences). The SGC-996 cell line was provided by Prof. Anonymize (Blinded Per Author Guidelines). The GBC-SD and SGC-996 cells were given 25 units/ml of penicillin, 25 g/ml of streptomycin, and 10% fetal bovine serum (FBS) (v/v) in a 5% (v/v) CO_2_ humidified incubator at 37 °C, and they were cultivated in DMEM and RPMI-1640, respectively. Our hospital provided the GBC tumor tissues and matched noncancerous tissues.

### RNA extraction and quantitative real-time PCR (qRT-PCR)

TRIzol reagent (Ambion, USA) was used to extract total RNA from cells, and miRNeasy FFPE kits (Qiagen, USA) were used to isolate the FFPE tissues based on the manufacturer’s protocols. All-in-One™ miRNA qRT-PCR Detection Kit (GeneCopoeia, USA) or Hifair® II 1st Strand cDNA Synthesis SuperMix (Yeasen, China) were used to make complementary DNA from 1 μg of RNA. qRT-PCR was performed using a LightCycler® 480 (Roche Molecular Systems, Inc., USA). The primers used for miRNA qRT-PCR were shown in Additional file 1: Table S1, and 5s small nuclear RNA was the miRNA assays internal control. Based on the cycle thresholds (CT), qRT-PCR results were examined and calculated as the relative RNA levels. The FC in RNA levels between each sample was examined by the 2^−ΔΔCT^ method.

### Cell transfection

Hsa-miRNA mimics and cognate negative control RNAs were obtained from RiBo (Guangzhou, China) and transfected into GBC cell lines at the concentration of 50 nM using Lipofectamine™ 3000 (Invitrogen, USA) based on the user instructions.

### Wound healing assay and transwell assay

After transfection, 5 × 10^4^ GBC cells were seeded in ibidi Culture-Insert (ibidi GmbH, Martinsried, Germany) on a 12-well plate. The Culture-Insert was gently removed after appropriate cell attachment. Then the cells could migrate into the wound area. Photomicrographs were taken with a microscope at 0 h and 48 h after wounding, respectively. The cell invasion assay was performed by 24-well Transwell chambers (Corning, USA). Next, 1 × 10^5^ cells were evenly suspended in a 200 µl serum-free medium and added to the up-layer inserts after transfection. 600 µl of medium with 20% FBS was then added to the low-layer compartment for chemoattractant. The cells on the membrane top surface were excised using a cotton-wool bud, whereas the cells on the bottom surface were fixed with 4% paraformaldehyde and stained with 0.1% crystal violet after 24-h incubation. Five random visual fields in each chamber were captured and counted under a microscope (Zeiss, German).

### Immunohistochemistry (IHC)

IHC was further conducted to indirectly validate the potential target genes expression of miR-642a-3p and miR-145-5p. Briefly, 4-μm sections from tissue blocks of the included GBC patients in our hospital were taken on coated slides. Then the slides were deparaffinized, hydrated, and blocked with hydrogen peroxide. The appropriate buffer for each antibody was utilized for heat induced antigen retrieval. The slides were incubated with primary antibody, following with secondary antibody (anti-*SYK* antibody, anti-*SH3GL1* antibody, anti-*CDKN1A* antibody, anti-*MYC* antibody, anti-*VEGFA* antibody, anti-*EGFR* antibody). Diaminobenzidine (DAB) chromogen and haematoxylin were used for staining and counterstaining, respectively.

### Statistical analysis

Unpaired tailed student’s *t* tests, Kaplan–Meier survival analysis, and log-rank tests were used for statistical analyses with GraphPad Prism 8 (GraphPad Software, Inc., La Jolla, CA, USA). The results were shown as mean ± standard deviation (SD). *P* < 0.05 was considered statistically significant.

## Results

### Identification of miRNAs of different expression and their target genes

**Fig. 1:**
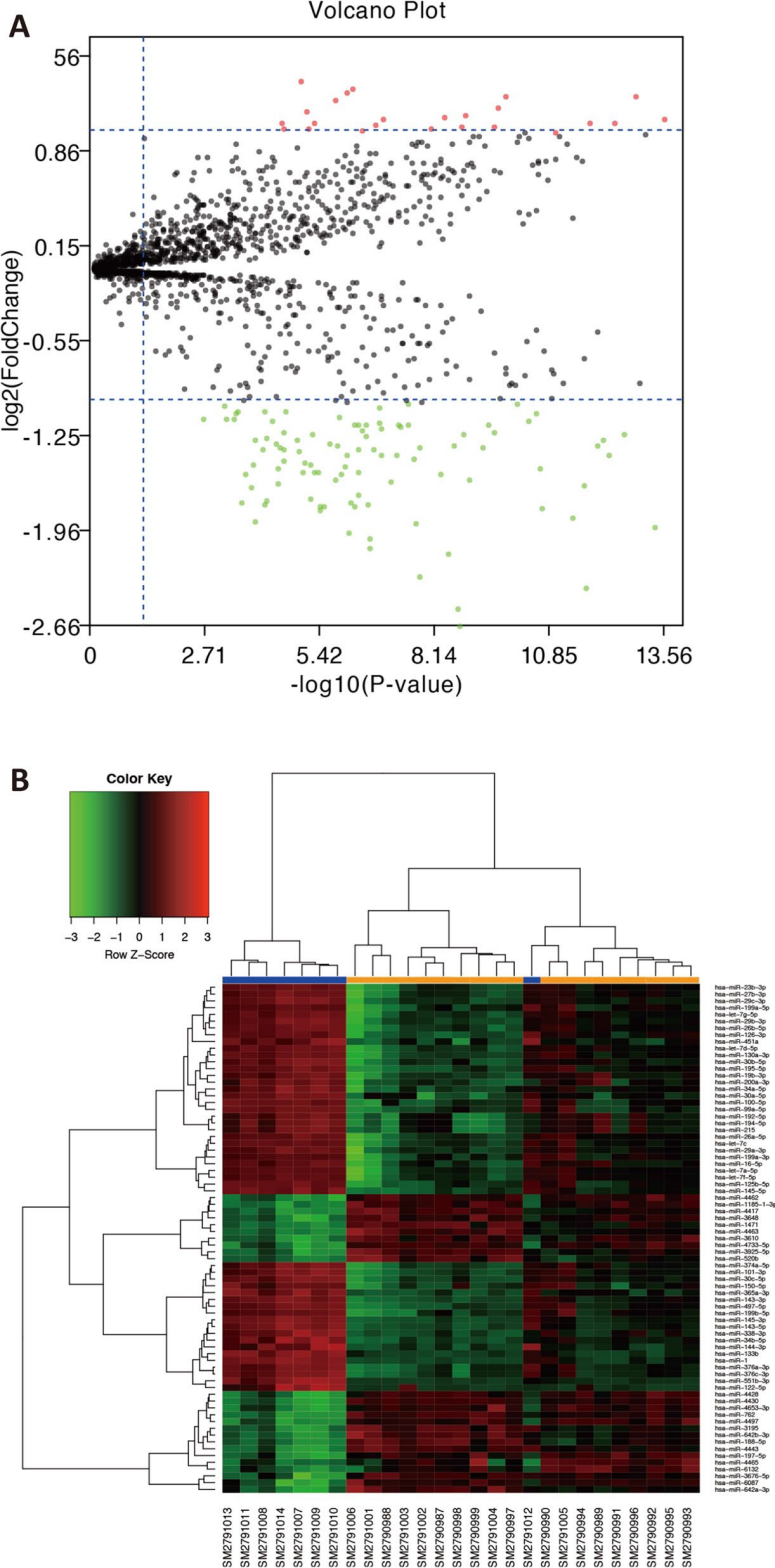
MiRNAs clustering of different expression. **A** Volcano plots of differentially expressed miRNAs. Green data points indicated down-regulated miRNAs and red data points indicated up-regulated miRNAs. The differences was set as |log FC|> 1. Black dots indicated miRNAs that were not differentially expressed. **B** The heatmap of differentially expressed miRNAs. Log FC of the genes was represented by the top-right legend. *FC* fold change

MiRNA array GSE104165 was retrieved from the GEO dataset (Additional file 1: Fig. S1). The volcano plot and heatmap with miRNAs clustering of different expression were portrayed in Fig. 1. A total of 122 miRNAs in GBC tumor samples were identified to be differentially expressed, among which the expression of 25 miRNAs was found to be significantly up-regulated and 97 miRNAs were down-regulated. The top 10 most up-regulated and down-regulated miRNAs were summarized in Tables 1 and 2, respectively. Then, MiRTarBase was applied to identify the possible target genes of the up- and down-regulated miRNAs. Notably, the 3^rd^ up-regulated miRNA, miR-3676-5p, did not successfully predict target genes in the database, thus the top 2 miRNAs, including miR-4430 and miR-642-3p, were selected for further analysis. Similarly, miR-451a and miR-145-5p were the top 2 down-regulated miRNAs. MiRTarBase was used to identify the 477 potential target genes from the 2 most up-regulated miRNAs and 268 genes from the 2 most down-regulated miRNAs.

**Table 1:** The most up-regulated expression of miRNAs in GBC

miRNA	logFC	AveExpr	t	P. value	adj.P.Val	B
hsa-miR-4430	1.560415197	2.69965023	13.8088015	1.38E−13	3.94E−11	21.12578004
hsa-miR-642a-3p	1.388882733	3.838242328	5.380473512	1.18E−05	6.02E−05	2.872762389
hsa-miR-3676-5p	1.328712158	4.479439939	6.483236311	6.68E−07	5.02E−06	5.740789951
hsa-miR-642b-3p	1.305137316	2.643572949	6.337901273	9.69E−07	6.77E−06	5.368095939
hsa-miR-4653-3p	1.274020361	2.896301402	10.02878504	1.71E−10	5.90E−09	14.03773895
hsa-miR-4733-5p	1.264661058	0.999736641	13.82584744	1.34E−13	3.94E−11	21.1541377
hsa-miR-4443	1.241770025	3.026350821	6.115673242	1.72E−06	1.10E−05	4.7945584
hsa-miR-4428	1.193376354	2.79367948	9.87297247	2.37E−10	7.80E−09	13.70803634
hsa-miR-4465	1.161175612	2.428359614	5.497611862	8.65E−06	4.56E−05	3.180798205
hsa-miR-3610	1.124733844	1.111994643	9.05744475	1.40E−09	3.34E−08	11.92953693

*GBC* gallbladder cancer, *FC* fold change

**Table 2.** The most down-regulated expression of miRNAs in GBC

miRNA	logFC	AveExpr	t	P. value	adj.P.Val	B
hsa-miR-451a	− 2.657515961	2.162795615	− 8.922076057	1.89E−09	4.22E−08	11.62565902
hsa-miR-145-5p	− 2.530888937	2.586220072	− 8.866540584	2.14E−09	4.73E−08	11.5002749
hsa-miR-1	− 2.378360609	0.335278283	− 12.25075546	2.14E−12	2.69E−10	18.40491209
hsa-miR-99a-5p	− 2.29346529	1.634070053	− 7.94855799	1.80E−08	2.48E−07	9.367532444
hsa-miR-143-3p	− 2.113226719	1.198615156	− 8.624341644	3.72E−09	7.33E−08	10.94857798
hsa-miR-29c-3p	− 2.069281379	2.228370391	− 6.848924471	2.64E−07	2.33E−06	6.669209031
hsa-miR-125b-5p	− 2.012061309	2.869352599	− 6.861116734	2.56E−07	2.30E−06	6.699917987
hsa-miR-195-5p	− 1.962473569	1.488055322	− 6.378651302	8.72E−07	6.16E−06	5.472794062
hsa-miR-133b	− 1.917864513	0.445914537	− 14.4344557	4.87E−14	3.26E−11	22.14778776
hsa-miR-100-5p	− 1.891570713	1.864235972	− 7.865181939	2.19E−08	2.88E−07	9.168268045

*GBC* gallbladder cancer, *FC* fold change

### GO functional annotation analysis

**Fig. 2:**
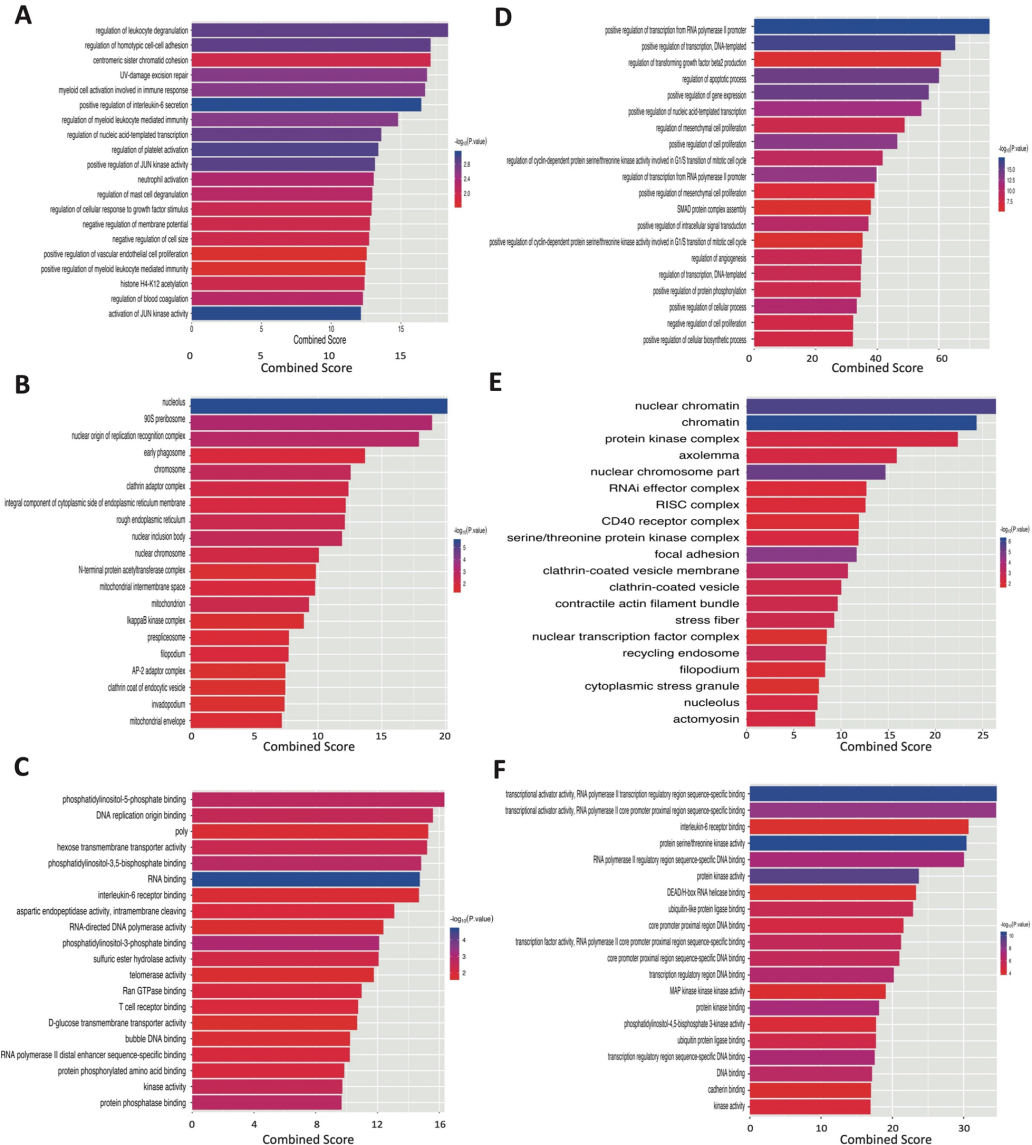
GO functions for the target genes of the up-regulated and down-regulated miRNAs. **A** Up-regulated miRNA enriched biological process. **B** Up-regulated miRNAs enriched cellular component. **C** Up-regulated miRNAs enriched molecular function. **D** Down-regulated miRNAs enriched biological process. **E** Down-regulated miRNAs enriched cellular component. **F** Down-regulated miRNAs enriched molecular function. *GO* gene ontology

Based on the potential target genes, 3 GO functional annotation analysis categories involving biological process (BP), cellular component (CC), and molecular function (MF) were conducted. The followings were enriched GO functions for the target genes of up-regulated miRNA: leukocyte degranulation, regulation of homotypic cell–cell adhesion, and centromeric sister chromatid cohesion in BP category; nucleolus, 90S pre-ribosome and nuclear origin of replication recognition complex in CC category; and phosphatidylinositol-5-phosphate binding, DNA replication origin binding and poly in MF category (Fig. 2A–C). The enriched GO functions for the 2 down-regulated miRNAs target genes were shown in Fig. 2D–F, involving the transcription from RNA polymerase II (RNAP II) promoter positive regulation, transcription positive reregulation, DNA-templated, transforming growth factor beta2 production in BP category regulation; nuclear chromatin, chromatin and protein kinase complex in CC category; and RNAP II transcription regulatory region sequence-specific binding, transcriptional activator activity, RNAP II core promoter proximal region sequence-specific binding and interleukin-6 receptor binding in MF category.

### Enrichment analysis in KEGG pathway

**Fig. 3:**
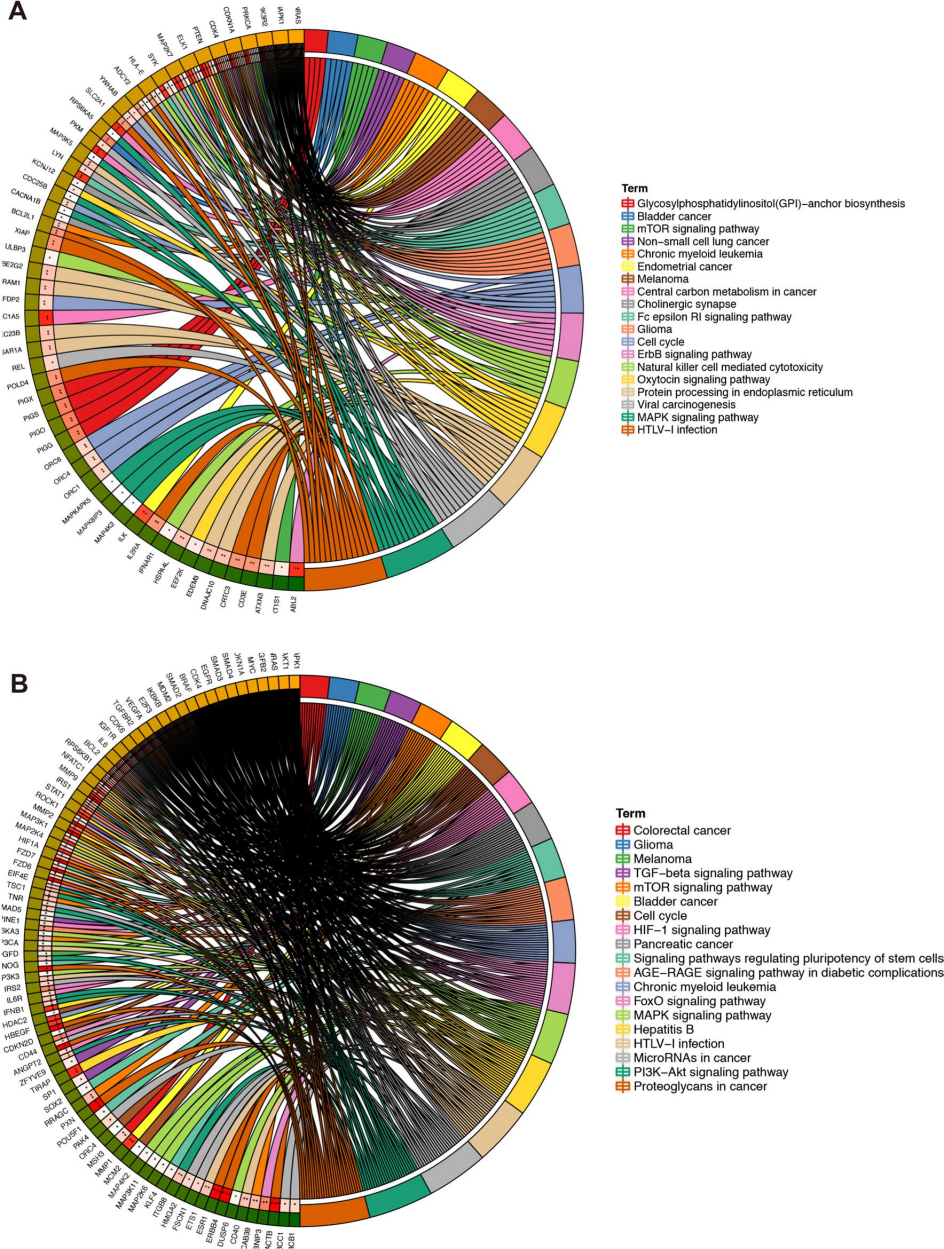
The distribution of target genes of four selected miRNAs of different expression in GO-enriched functions. **A** Up-regulated miRNAs and (**B**) down-regulated miRNAs. Symbols of target genes were displayed on the left side of the graph. Gene involvement under GO terms was established by the colored connecting lines to the right. *GO* gene ontology. *P < 0.05; **P < 0.01

We subsequently performed a KEGG pathway enrichment analysis to investigate the target genes enriched pathways. As shown in Fig. 3A, the enriched KEGG pathways for up-regulation were in HTLV-1 infection, MAPK signaling pathway, and viral carcinogenesis. The enriched KEGG for down-regulated miRNAs included proteoglycans and microRNA in cancer, and the PI3K-Akt signaling pathway (Fig. 3B). mTOR pathway plays the central role in tumor progression of GBC [20, 21]. Therefore, 5 genes (*AKT1S1, PTEN, MAPK1, PIK3R2, PRKCA*) of up-regulated miRNAs and 13 genes (*CAB39, IRS1, BRAF, TSC1, HIF1A, VEGFA, IKBKB, RPS6KA3, RRAGC, RPS6KB1, AKT1, MAPK1, EIF4E*) of down-regulated miRNAs, which were involved in mTOR pathway and interacted with the selected miRNAs, may be associated with GBC progression.

### MiRNA-hub and PPI network construction

STRING online database was applied to generate a PPI network, based on the 4 selected miRNAs (top 2 most up-regulated and top 2 most down-regulated) with different expression as well as potential target genes. The top 10 hub genes were screened out according to node degrees (Table 3). The hub genes for the up-regulated miRNAs were identified as *MARPK1, PTEN, SYK, TAF1, CDKN1A, POLR2E, PRKCA, CDK4, SH3GL1*, and *DNAJC10*. Meanwhile, the hub genes for the down-regulated miRNAs were *AKT1, VEGFA, MAPK1, EGFR, MYC, IL6, BCL2, ACTB, SMAD4, ESR1.*

**Table 3.** Identification of hub genes by PPI network

Up-regulated miRNAs		Down-regulated miRNAs	
Gene	Degree	Gene	Degree
*MAPK1*	34	*AKT1*	76
*PTEN*	27	*VEGFA*	68
*SYK*	25	*MAPK1*	65
*TAF1*	23	*EGFR*	63
*CDKN1A*	22	*MYC*	59
*POLR2E*	21	*IL6*	57
*PRKCA*	21	*BCL2*	52
*CDK4*	19	*ACTB*	44
*SH3GL1*	19	*SMAD4*	43
*DNAJC10*	18	*ESR1*	42

*PPI* protein–protein interaction

**Fig. 4:**
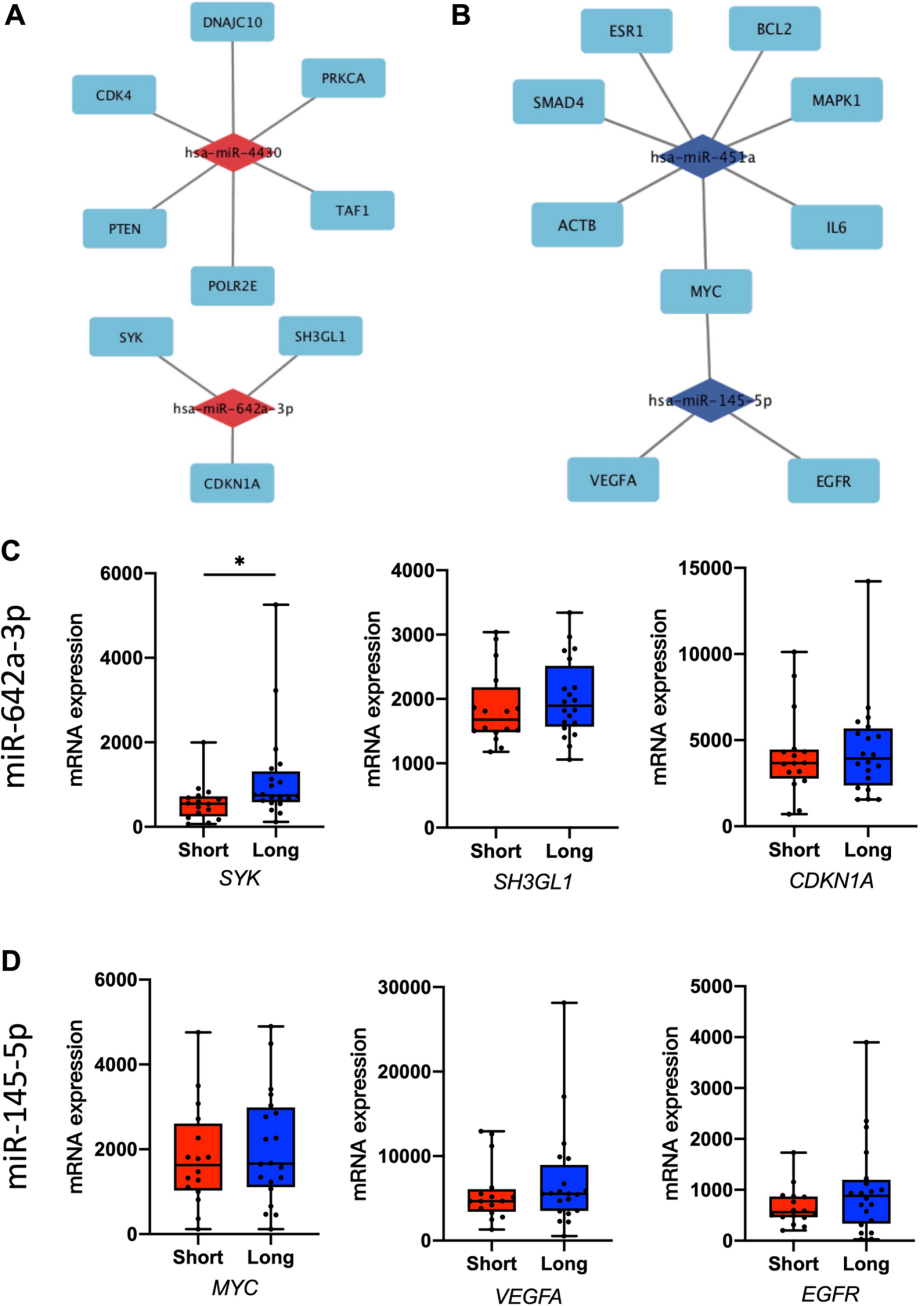
The regulatory network between dysregulated miRNAs and hub genes. **A** Up-regulated miRNAs and (**B**) down-regulated miRNAs. The mRNA expression of predicted targets for miRNAs were from the TCGA database. **C** MiR-642a-3p: *SYK* expression, *SH3GL1* expression, *CDKN1A* expression. **D** MiR-145-5p: *MYC* expression, *VEGFA* expression, *EGFR* expression. *P < 0.05

Cytoscape software was utilized to construct the miRNA-hub gene network. We found that 6 hub genes (*CDK4, DNAJC10, PRKCA, TAF1, POLR2E,* and *PTEN*) could be regulated by up-regulated miR-4430, and 3 hub genes (*SYK, SH3GL1,* and *CDKN1A*) could be regulated by miR-642a-3p (Fig. 4A). In addition, miR-451a could potentially target 7 genes (*SMAD4, ESR1, BCL2, MARPK1, IL6, MYC,* and *ACTB*), and 3 hub genes (*MYC, VEGFA,* and *EGFR*) could be regulated by miR-145-5p (Fig. 4B).

### Validation of potential target genes using TCGA database

Next, we evaluated the potential targets of hub genes with the TCGA database. Based on short and long survival, *SYK* and *SH3GL1*, but *CDKN1A*, had a similar trend of overall survival like miR-642a-3p (Fig. 4C). The further analysis showed *CDKN1A* had a similar result with miR-642a-3p based on different T stages, which represented tumor invasion (Additional file 1: Fig. S2A). In the meantime, *MYC* and *VEGFA*, like miR-145-5p, were related to T stages (invasion-related) (Additional file 1: Fig. S2B-C; Fig. 4D).

### Prognostic roles of miR-642a-3p and miR-145-5p and validation of predicted hub genes using TCGA database and SRRSH database

**Fig. 5:**
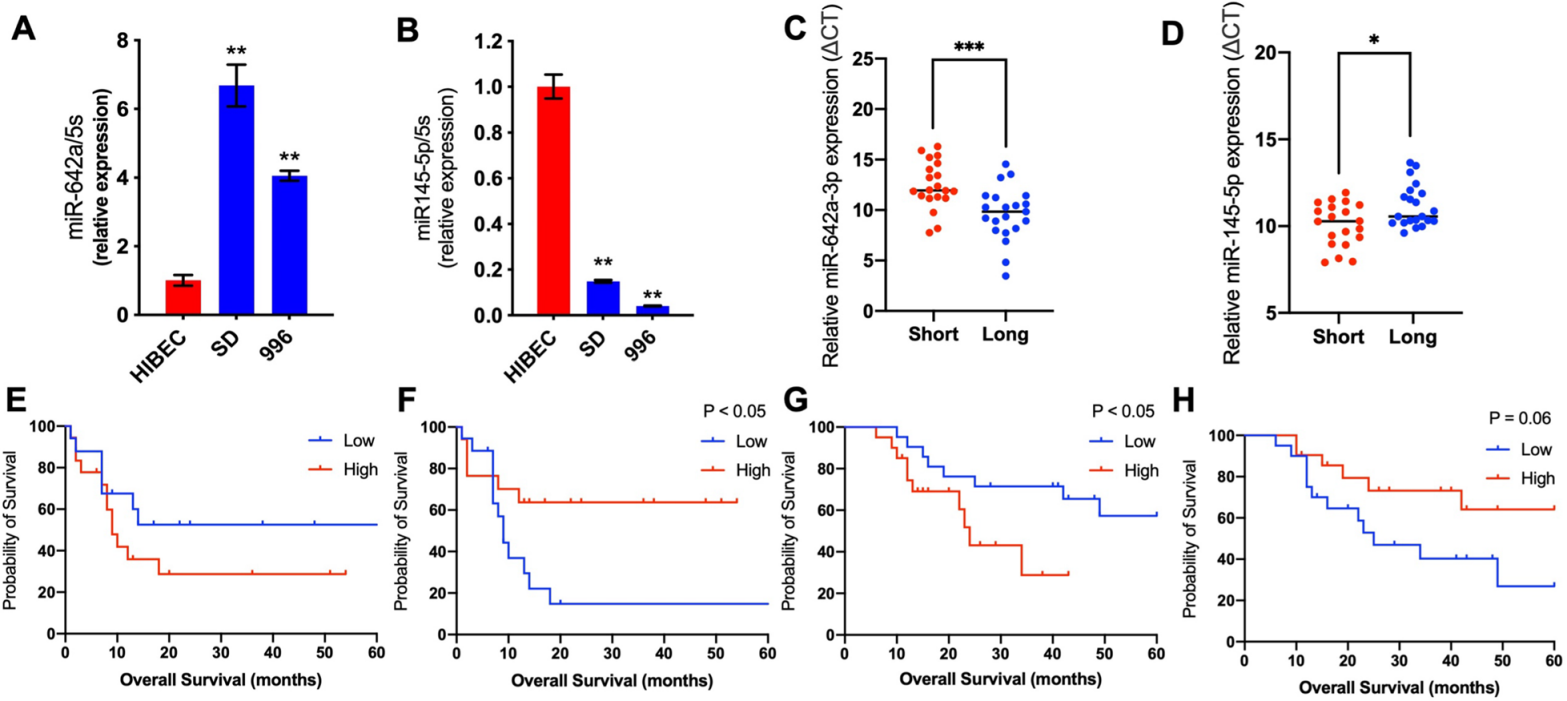
The expression and prognostic roles of miR-642a-3p and miR-145-5p in GBC. **A** MiR-642a-3p expression and (**B**) miR-145-5p expression in 2 GBC cell lines (SGC-996, GBC-SD) were compared with HiBEC. **C** The expression of miR-642a-3p and (**D**) miR-145-5p in tumor tissues of GBC patients with different prognosis. **E** Kaplan–Meier curve of OS in CHOL patients with high vs. low expression of miR-642a-3p from TCGA database. **F** Kaplan–Meier curve of OS in CHOL patients with high vs. low expression of miR-145-5p for patients from TCGA database. **G** Kaplan–Meier curve of OS with high vs. low expression of miR-642a-3p in GBC patients from clinical data. **H** Kaplan–Meier curve of OS with high vs. low expression of miR-145-5p in GBC patients from clinical data. *GBC* gallbladder cancer, *OS* overall survival, *CHOL* cholangiocarcinoma, *TCGA* the Cancer Genome Atlas. *P < 0.05; **P < 0.01; ***P < 0.001; ****P < 0.0001

Further investigation was applied to analyze the expression and clinical significance of both miR-642-3p and miR-145-5p in GBC. GBC-SD and SGC-996 cell lines, primary GBC tissues samples were detected for the expression of miR-642-3p and miR-145-5p. It was confirmed that miR-642-3p were up-regulated and miR-145-5p were down-regulated in GBC cell lines (Fig. 5A, B). Using SRRSH database, we found that miR-642-3p and miR-145-5p were respectively up-regulated and down-regulated in GBC patients with poor prognosis via RNA extraction and qRT-PCR based on their tissue slides (Fig. 5C, D). IHC was further conducted to indirectly validate the potential target genes expression of miR-642a-3p and miR-145-5p. Similar results were observed in related target genes, such as *SYK*, *SH3GL1*, *CDKN1A*, *MYC*, *VEGFA* and so on (Additional file 1: Fig. S3).

Survival analysis was performed to assess the prognostic value of miR-642-3p and miR-145-5p in GBC. Although miR-642-3p was not associated with worse OS in TCGA (Fig. 5E), high miR-642-3p presented decreased survival in our hospital data (Fig. 5G). It was implied that miR-642-3p may be a potential oncogene. By contrast, miR-145-5p was indicated as a favorable prognostic factor both in TCGA (Fig. 5F) and our data (Fig. 5H). Collectively, these data indicated that miR-642-3p and miR-145-5p were unusually expressed, which could be prognostic biomarkers and associated with invasion and metastasis in GBC.

### *Validation of potential target genes of miR-642a-3p and miR-145-5p *in vitro

We used miR-642a-3p inhibitor and miR-145-5p mimics for further validation of potential target genes of miR-642a-3p and miR-145-5p, respectively. When using miR-642a-3p inhibitor, we found mRNA level of *SH3GL1* and *CDKN1A* were decreased (Additional file 1: Fig. S4A). Moreover, when using miR-145-5p mimics, we found mRNA level of *MYC*, *VEGFA*, and *EGFR* were significantly regulated (Additional file 1: Fig. S4B). Besides, the relationship of the above-mentioned genes with tumor invasion have been reported by previous studies [22–25].

### *MiR-642a-3p and miR-145-5p on GBC migration and invasion *in vitro

**Fig. 6:**
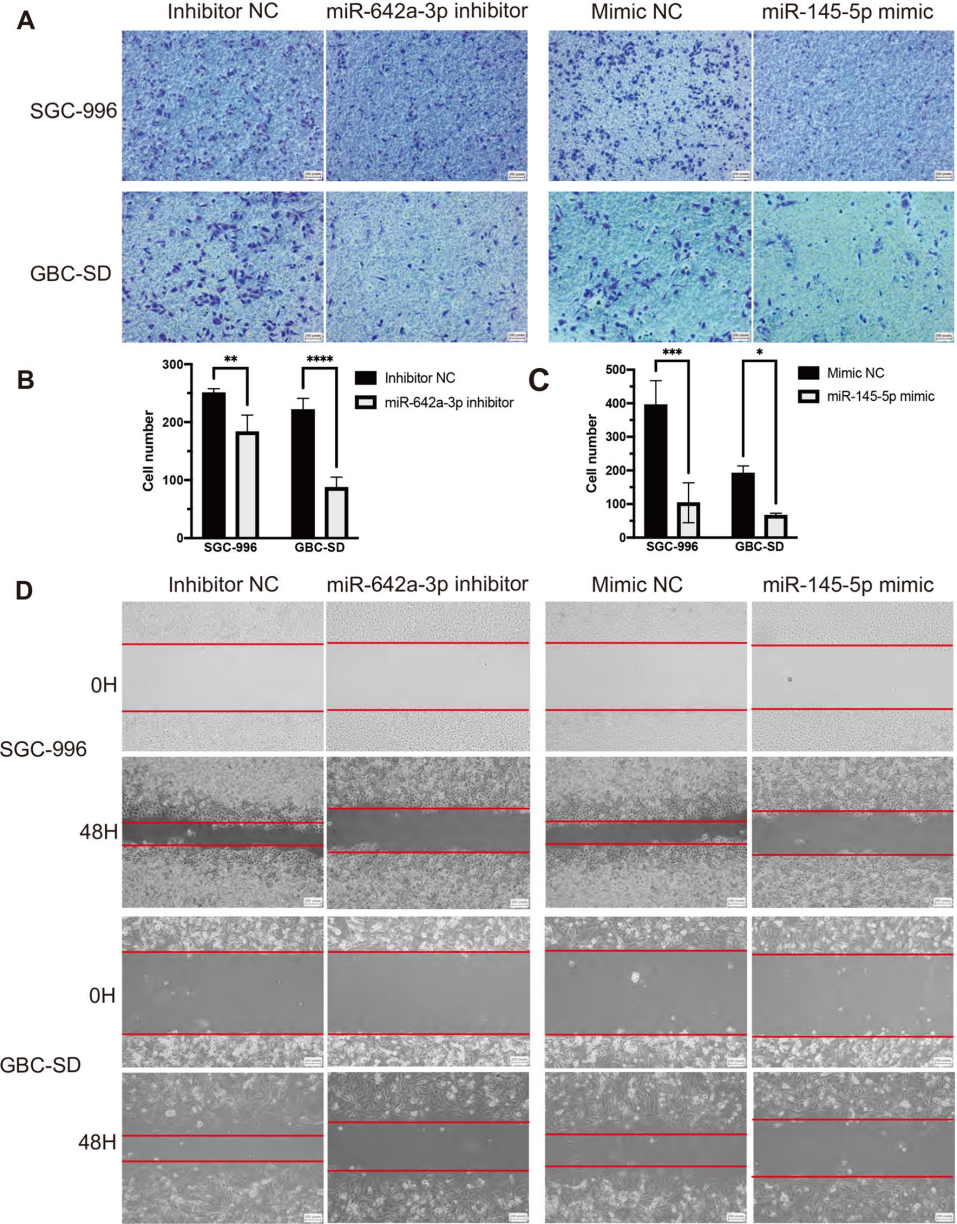
MiR-642a-3p under-expression and miR-145-5p over-expression regulated GBC invasion and migration. **A**–**C** After being transfected with miR-642a-3p inhibitor and miR-145-5p mimic, SGC-996 and GBC-SD showed weaker invasion than the control cells, respectively. **D** SGC-996 and GBC-SD were transfected with inhibitor NC, miR-642a-3p inhibitor, mimic NC, miR-145-5p mimic, respectively. Wound healing assay was performed in GBC cell with 48 h of recovery. GBC: Gallbladder cancer. *P < 0.05; **P < 0.01; ***P < 0.001; ****P < 0.0001. Error bars represented SD for n = 3

To examine the role of miR-642a-3p and miR-145-5p in GBC migration, a wound-healing assay was employed. RT-qPCR revealed significantly decreased miR-642a-3p and increased miR-145-5p levels of GBC cell lines after transfection, respectively (Additional file 1: Fig. S5). Our results demonstrated that decreased miR-642a-3p expression and increased miR-145-5p expression markedly suppress the migration ability of GBCs compared to negative control cells (Fig. 6A). Transwell invasion assay was performed to evaluate the influence of both miRNAs on GBC invasion. The results demonstrated the decrease of miR-642a-3p and over-expressed miR-145-5p suppressed cell invasion, respectively (P < 0.05, Fig. 6B–D). Hence, all data confirmed that both 2 miRNAs (miR-642a-3p and miR-145-5p) were key regulators relevant to invasion and metastasis of GBC, and approaches of targeting them may represent novel directions to improve the GBC prognosis.

## Discussion

In the study, miR-642a-3p and miR-145-5p were identified as invasion-metastasis associated miRNAs according to the bioinformatic analysis and experimental validation. As both two miRNAs were crucial in GBC invasion and metastasis, they would be utilized as promising targets for effective treatment to improve the prognosis of GBC patients.

Currently, miRNAs were gradually considered as essential regulators for tumor initiation, promotion, and progression transcriptional dynamics in GBC [26, 27], and thus researchers should pay more attention to systemically analyzing the invasion-metastasis associated miRNA in GBC. MiR-7-2-3p and miR-29c-3p were identified as metastasis suppressors for GBC via high-throughput screening, and they were also related to the pathogenesis of GBC [28]. Meanwhile, Ma et al*.* [29] demonstrated that miR-663a could regulate epithelial membrane protein-3 to suppresses GBC progression via interfering the MAPK/ERK pathway, indicating that the miR-663a/EMP3/MAPK/ERK axis would be potential treatment for GBC. Besides, after being activated by TGF-β1, miR-20a could play a crucial role in the pathogenesis and trigger metastasis of GBC through the miR-20a/Smad7/β-catenin axis [26]. Despite the identified miRNAs, further studies would be performed to offer more insights into improving the prognosis of GBC.

Indeed, a total of 4 miRNAs, including the top 2 up-regulated miRNAs (miR-642a-3p and miR-4430) and the top 2 down-regulated miRNAs (miR-145-5p and miR-451a), were selected for experimental validation. It was shown that miR-145-5p up-regulation and miR-642a-3p down-regulation regulated cell invasion and migration in GBC. Interestingly, the decrease in miR-4430 expression suppressed cell invasion and wound healing migration to enhance GBC loss-of-function, in the meantime, up-regulation of miR-451a significantly suppressed the invasion (Additional file 1: Fig. S6). Similar to our results, Ueta et al*.* [30] found that serum EVs miR-451a were significantly down-regulated and miR-451a inhibited GBC cell proliferation and induced apoptosis. Taken together, miR-451a would also be a novel therapeutic target for GBC patients.

In the miRNA-gene hub network, the hub genes were potentially regulated by miR-145-5p and miR-642a-3p. Various studies demonstrated that miR-145-5p was linked with various types of cancer, such as hepatocellular carcinoma [31], gastric cancer [32, 33], and upper tract urothelial carcinoma [34]. MiR-642a-3p was also linked to cell migration and invasion of hepatocellular carcinoma [35]. Besides, miR-29c-3p was found to be down-regulated (Table 2), which was in accordance with the results that up-regulation of miR-29c-3p could reverse EMT and decrease the metastasis ability in vitro and in vivo [28]. Similar to our results in Table 2, Jin et al*.* [36] also found that miR-143-3p suppressed tumor angiogenesis and growth of GBC through the ITGA6/PI3K/AKT/PLGF pathways. The aberrant expression of miRNAs have been relevant to GBC tumorigenesis and progression.

qRT-PCR results indicated that miR-145-5p and miR-642a-3p were significantly up- and down-regulated among GBC cell lines and clinical samples, respectively. MiR-145-5p up-regulation and miR-642a-3p down-regulation could significantly suppress in vitro activation, migration, and invasion of GBC using wound-healing and Transwell invasion assay. To explore the possible pathways, we performed GO annotation and KEGG pathway analysis for the predicted target genes of the top 2 most up- and down-regulated miRNAs using the Enrichr tool. Several target genes were revealed to be enriched in cell–cell adhesion, which was closely correlated to cell migration and invasion. Therefore, it would be a promising mechanism that can relieve the impacts of miR-145-5p and miR-642a-3p for GBC by targeting these genes.

The study has several limitations that need to be addressed. First, the public dataset with both miRNA and mRNA profiling in GBC was limited. Therefore, we tried to validate our results using different GBC databases (TCGA and SRRSH databases). Unfortunately, some verification results would not be obvious because of the small sample size. And further validation experiments should be performed with a large sample size. Second, merely 2 most up- and down-regulated miRNAs and the relevant target genes were included in the enrichment analysis. Further studies would be focused on more miRNAs of different expression to explore more potential treatment for GBC. Third, we selected 2 up- and down-regulated miRNAs for in vitro experiment validation, which would lead to the omittance of some functional miRNAs. And in vitro and in vivo experiments should be performed to reveal other functional miRNAs with a high validation accuracy. Finally, the deeper molecular mechanisms of GBC invasion and metastasis should also be explored in the future.

## Conclusions

In the present study, miR-642a-3p and miR-145-5p were identified as invasion-metastasis associated miRNAs via bioinformatic analysis and experimental validation. Both miR-642a-3p (down-regulation) and miR-145-5p (up-regulation) would be served as potential therapeutic targets for GBC in the future.

## Supplementary Information

**Additional file 1: Table S1**. List of the primers used for miRNA quantitative real-time PCR. Figure S1. Data GSE104165 was shown. Figure S2. The comparison of mRNA expression of hub genes based on different T stages. (A) MiR-642a-3p: CDKN1A expression. (B) MiR-145-5p: MYC expression. (C) MiR-145-5p: VEGFA expression. *P. Figure S3. The comparison of immunohistochemistry of GBC tissue samples based on different survival. (A) SYK. (B) SH3GL1. (C) CDKN1A. (D) MYC. (E) VEGFA. (F) EGFR. GBC: Gallbladder cancer. *P. Figure S4. The mRNA expression of predicted hub genes in GBC-SD cell. (A) The expression of SH3GL1, CDKN1A, and SYK in negative control vs. miR-642a-3p inhibitor. (B) The expression of MYC, EGFR, and VEGFA in negative control vs. miR-145-5p mimics. GBC: Gallbladder cancer. *P. Figure S5. (A-B) After being transfected with 50 nM mimics, the expression of miR-642a-3p and miR-145-5p were significantly elevated in SGC-996 and GBC-SD cell lines. qRT-PCR was applied to detect the miR-642a-3p and miR-145-5p expression levels after 48h transfection. GBC: Gallbladder cancer; qRT-PCR: Quantitative real-time PCR. *P . Figure S6. Down-regulation of miR-4430 and up-regulation of miR-451a regulated GBC cell invasion and metastasis. (A) SGC-996 and GBC-SD, which were transfected with miR-4430 inhibitor and miR-451a mimic, invaded less versus control cancer cells, respectively. (B) Quantification of SGC-996 and GBC-SD after miR-4430 inhibitor. (C) Quantification of SGC-996 and GBC-SD after miR-451a. (D) SGC-996 and GBC-SD were transfected with inhibitor NC, miR-4430 inhibitor, mimic NC, miR-451a mimic, respectively. Wound healing assay was performed in GBC cell with 48h of recovery. GBC: Gallbladder cancer. NC: Negative control. ***P.

**Additional file 2**. Ethics file.

## Data Availability

The original data of the study are available from the corresponding authors upon reasonable request.

## Abbreviations

GBC: Gallbladder cancer
BTC: Biliary tract cancer
AJCC: American Joint Committee on Cancer
EMT: Epithelial–mesenchymal transition
PD-L1: Programmed cell death-ligand 1
NCBI: National Center for Biotechnology Information
GEO: Gene Expression Omnibus
FC: Fold change
GO: Gene Ontology
KEGG: Kyoto Encyclopedia of Genes and Genomes
PPI: Protein–protein interaction
TCGA: The Cancer Genome Atlas
OS: Overall survival
HiBEC: Human intrahepatic biliary epithelial cells
FBS: Fetal bovine serum
qRT-PCR: Quantitative real-time PCR
CT: Cycle thresholds
IHC: Immunohistochemistry
DAB: Diaminobenzidine
SEM: Standard error of mean
BP: Biological process
CC: Cellular component
MF: Molecular function
RNAP II: RNA polymerase II
