# Supplementary material for: Identification of invasion-metastasis associated MiRNAs in gallbladder cancer by bioinformatics and experimental validation
Source: J Transl Med. 2022 Apr 28;20:188. doi: 10.1186/s12967-022-03394-8 (PMC9052523; doi:10.1186/s12967-022-03394-8)
Supplement: Supplementary file 1 — Additional file 1: Table S1. List of the primers used for miRNA quantitative real-time PCR. Figure S1. Data GSE104165 was shown. Figure S2. The comparison of mRNA expression of hub genes based on different T stages. (A) MiR-642a-3p: CDKN1A expression. (B) MiR-145-5p: MYC expression. (C) MiR-145-5p: VEGFA expression. *P. Figure S3. The comparison of immunohistochemistry of GBC tissue samples based on different survival. (A) SYK. (B) SH3GL1. (C) CDKN1A. (D) MYC. (E) VEGFA. (F) EGFR. GBC: Gallbladder cancer. *P. Figure S4. The mRNA expression of predicted hub genes in GBC-SD cell. (A) The expression of SH3GL1, CDKN1A, and SYK in negative control vs. miR-642a-3p inhibitor. (B) The expression of MYC, EGFR, and VEGFA in negative control vs. miR-145-5p mimics. GBC: Gallbladder cancer. *P. Figure S5. (A-B) After being transfected with 50 nM mimics, the expression of miR-642a-3p and miR-145-5p were significantly elevated in SGC-996 and GBC-SD cell lines. qRT-PCR was applied to detect the miR-642a-3p and miR-145-5p expression levels after 48h transfection. GBC: Gallbladder cancer; qRT-PCR: Quantitative real-time PCR. *P . Figure S6. Down-regulation of miR-4430 and up-regulation of miR-451a regulated GBC cell invasion and metastasis. (A) SGC-996 and GBC-SD, which were transfected with miR-4430 inhibitor and miR-451a mimic, invaded less versus control cancer cells, respectively. (B) Quantification of SGC-996 and GBC-SD after miR-4430 inhibitor. (C) Quantification of SGC-996 and GBC-SD after miR-451a. (D) SGC-996 and GBC-SD were transfected with inhibitor NC, miR-4430 inhibitor, mimic NC, miR-451a mimic, respectively. Wound healing assay was performed in GBC cell with 48h of recovery. GBC: Gallbladder cancer. NC: Negative control. ***P. [file 12967_2022_3394_MOESM1_ESM.docx]

**Supplementary Materials:**

| **Table S1.** List of the primers used for miRNA quantitative real-time PCR. | |
| --- | --- |
| Gene | Sense |
| miRNA-4430 | ATAAAGGCTGGAGTGAGCGGAG |
| miRNA-642a-3p | ATATAAATTAGACACATTTGGAGAGGGAA |
| miRNA-451a | AATAAATAAATATAAACCGTTACCATTACTGAG |
| miRNA-145-5p | AATAAGTCCAGTTTTCCCAGGAATC |
| 5s | GGGAATACCGGGTGCTGTAGGCT |
| The primers were purchased from Tsingke. | |

**
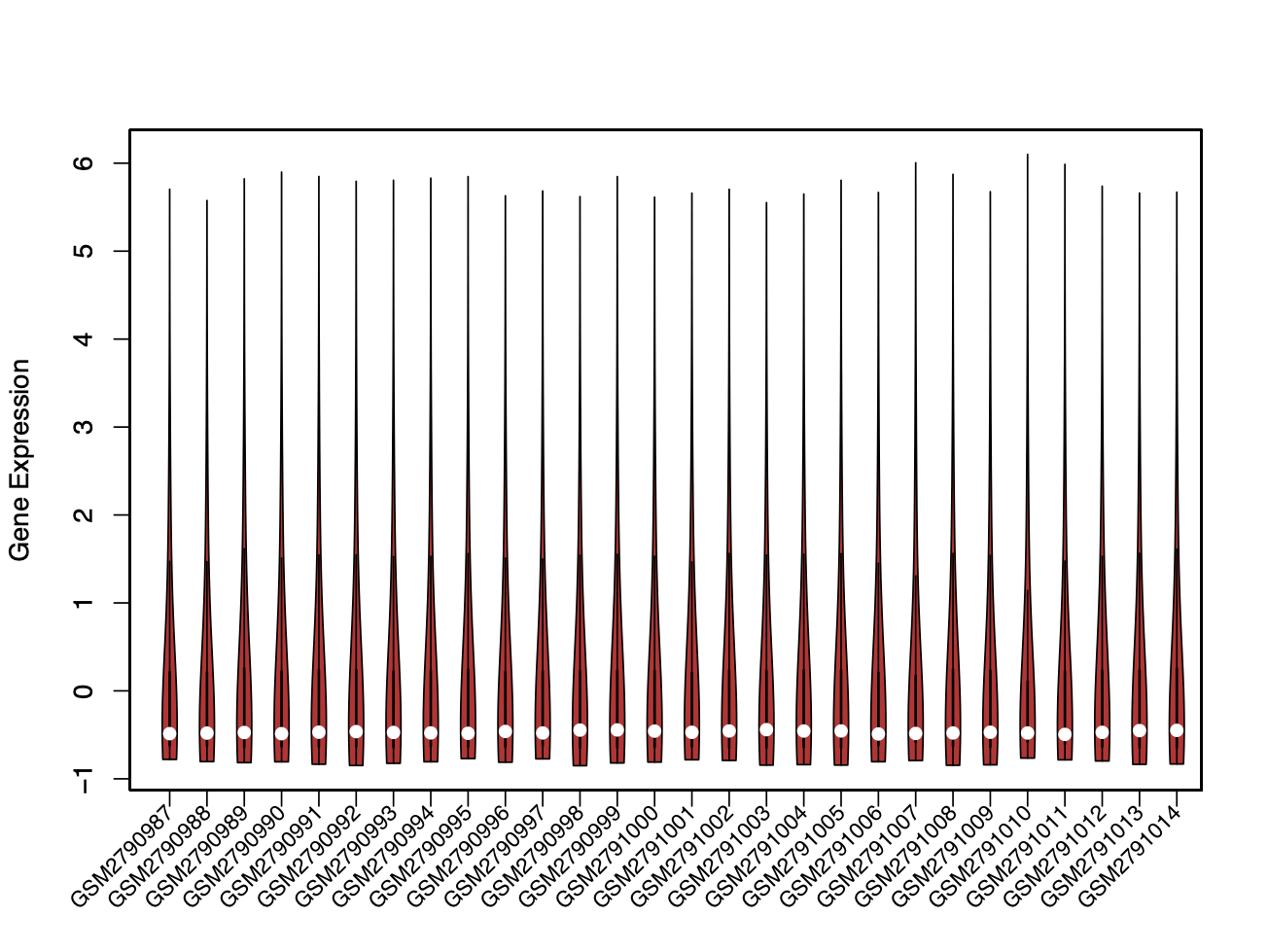
**

**Figure S1.** Data GSE104165 was shown.


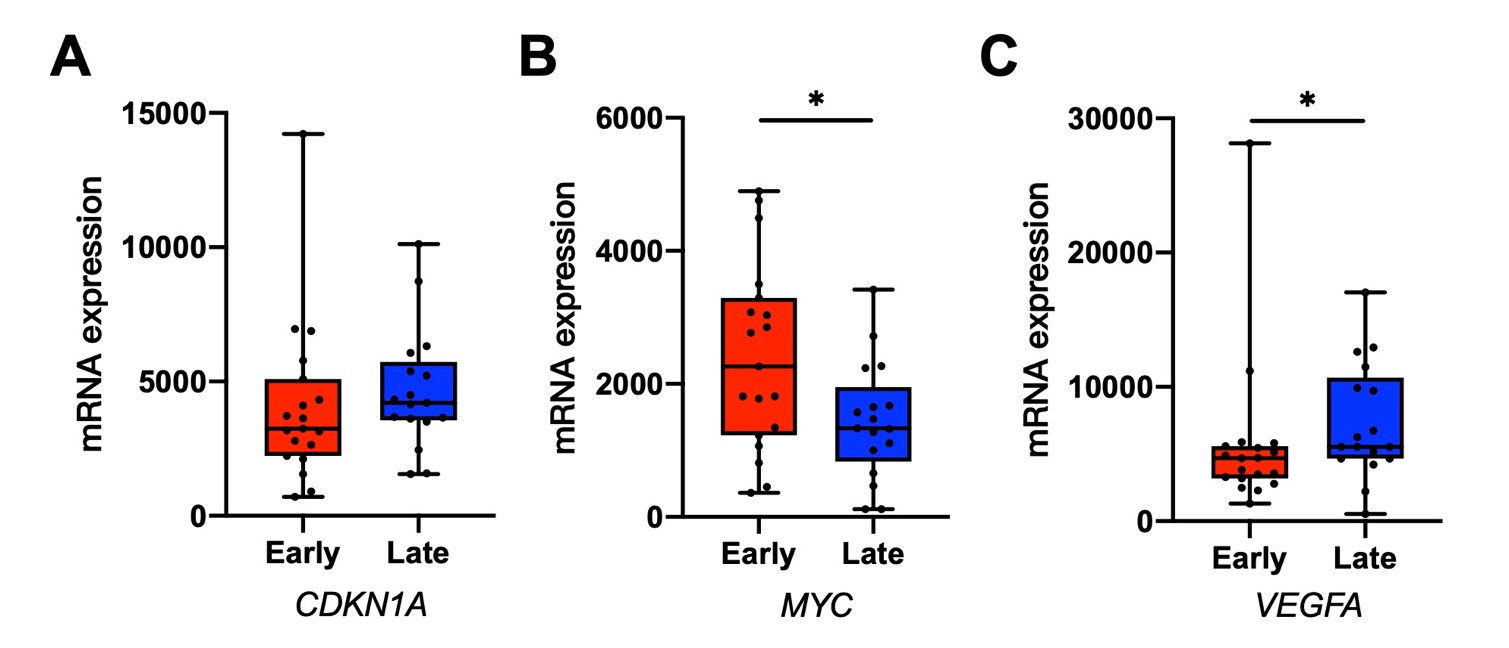


**Figure S2.** The comparison of mRNA expression of hub genes based on different T stages. (A) MiR-642a-3p: *CDKN1A* expression. (B) MiR-145-5p: *MYC* expression. (C) MiR-145-5p: *VEGFA* expression. **P* < 0.05.


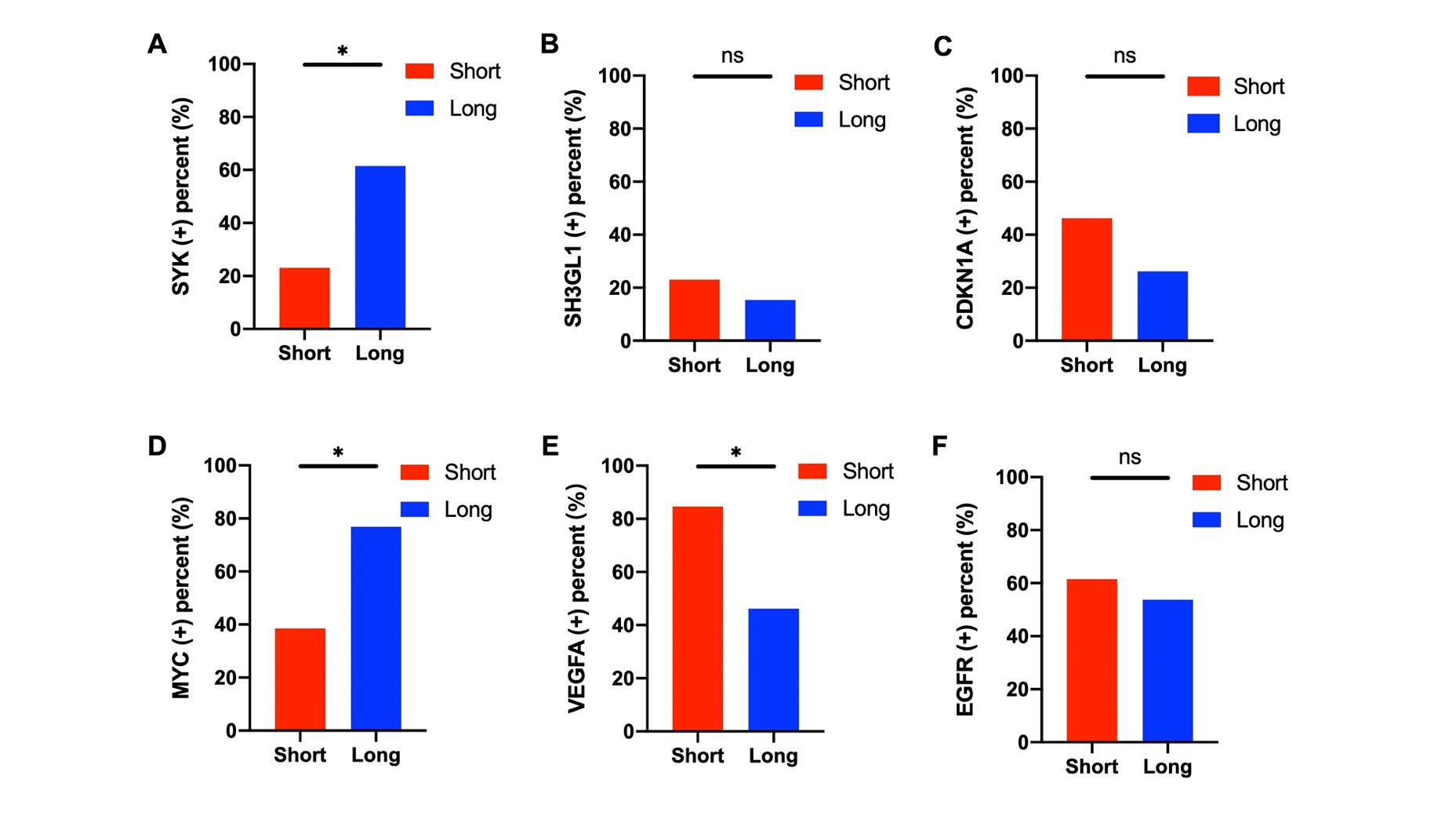


**Figure S3.** The comparison of immunohistochemistry of GBC tissue samples based on different survival. (A) SYK. (B) SH3GL1. (C) CDKN1A. (D) MYC. (E) VEGFA. (F) EGFR. GBC: Gallbladder cancer. **P* < 0.05.

**
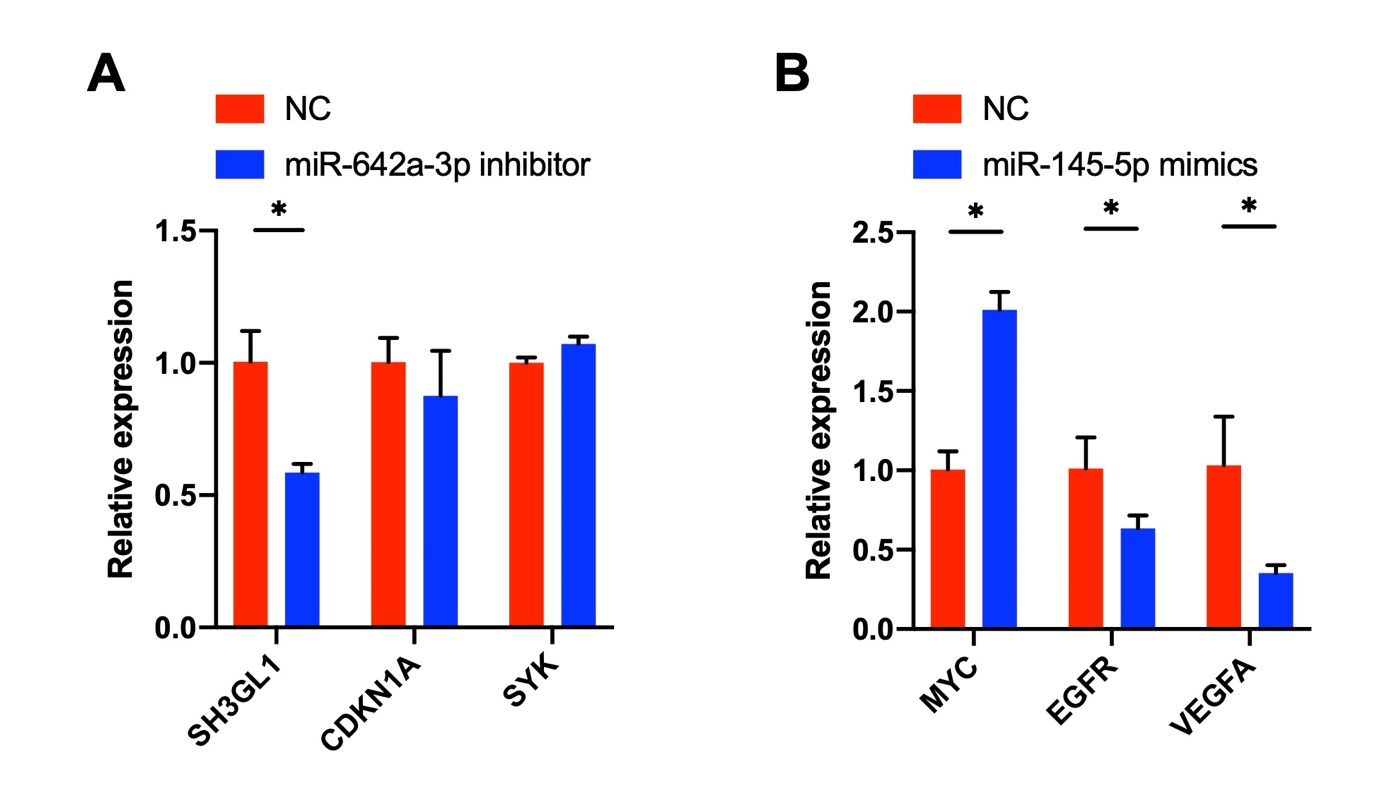
**

**Figure S4.** The mRNA expression of predicted hub genes in GBC-SD cell. (A) The expression of *SH3GL1*, *CDKN1A*, and *SYK* in negative control *vs.* miR-642a-3p inhibitor. (B) The expression of *MYC*, *EGFR*, and *VEGFA* in negative control *vs.* miR-145-5p mimics. GBC: Gallbladder cancer. **P* < 0.05.


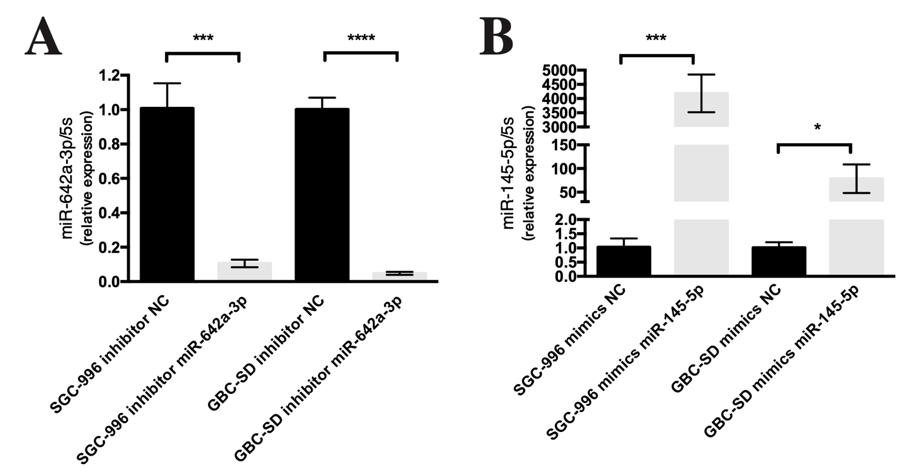


**Figure S5.** (A-B) After being transfected with 50 nM mimics, the expression of miR-642a-3p and miR-145-5p were significantly elevated in SGC-996 and GBC-SD cell lines. qRT-PCR was applied to detect the miR-642a-3p and miR-145-5p expression levels after 48h transfection. GBC: Gallbladder cancer; qRT-PCR: Quantitative real-time PCR. **P* < 0.05; ****P* < 0.001; **** *P* < 0.0001. Error bars represented SD for n = 3.


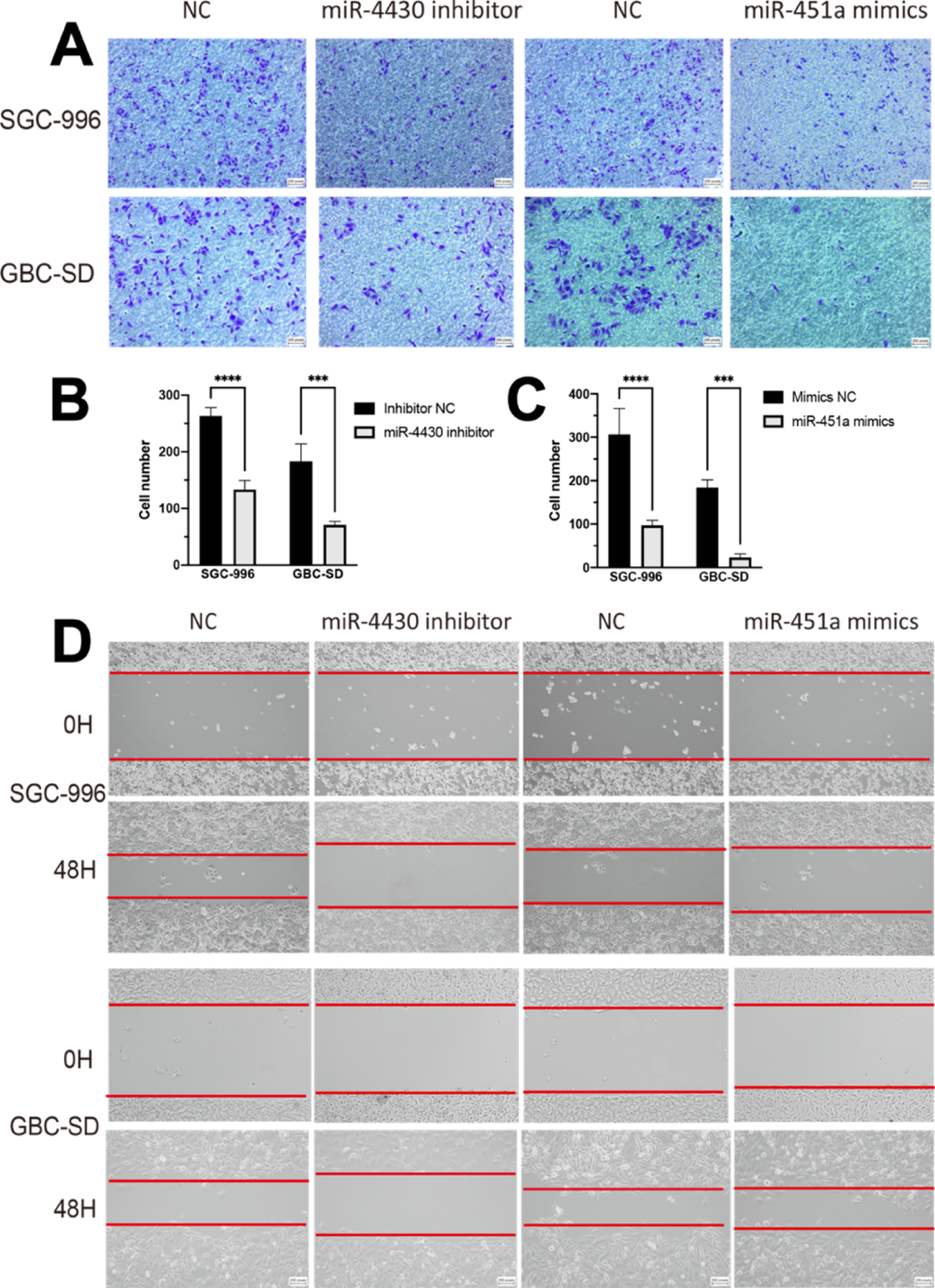


**Figure S6.** Down-regulation of miR-4430 and up-regulation of miR-451a regulated GBC cell invasion and metastasis. (A) SGC-996 and GBC-SD, which were transfected with miR-4430 inhibitor and miR-451a mimic, invaded less versus control cancer cells, respectively. (B) Quantification of SGC-996 and GBC-SD after miR-4430 inhibitor. (C) Quantification of SGC-996 and GBC-SD after miR-451a. (D) SGC-996 and GBC-SD were transfected with inhibitor NC, miR-4430 inhibitor, mimic NC, miR-451a mimic, respectively. Wound healing assay was performed in GBC cell with 48h of recovery. GBC: Gallbladder cancer. NC: Negative control. ****P* < 0.001; *****P* < 0.0001. Error bars represented SD for n = 3.
