# Supplementary material for: Identification of invasion-metastasis associated MiRNAs in gallbladder cancer by bioinformatics and experimental validation
Source: J Transl Med. 2022 Apr 28;20:188. doi: 10.1186/s12967-022-03394-8 (PMC9052523; doi:10.1186/s12967-022-03394-8)
Supplement: Supplementary file 2 — Additional file 2. Ethics file. [file 12967_2022_3394_MOESM2_ESM.pdf]

浙江大学医学院附属邵逸夫医院医学伦理委员会 快速伦理批件

批件号：科研 20210625-32

项目名称：通过生物信息学筛选胆囊癌中与侵袭转移相关的 microRNA 并进行体外验证	
主要研究者：陈鸣宇	申请单位：浙江大学医学院附属邵逸夫医院普外科
有效期：1 年	跟踪审查频率：12 个月
审查类别：■初始审查 □复审 □修正案审查 □年度跟踪审查	
审查文件：<br>1、初始审查申请表<br>2、临床研究项目负责人承诺书<br>3、研究方案（版本号：V1.0,版本日期：2021-06-06）<br>4、知情同意书（版本号：V1.0,版本日期：2021-06-06）<br>5、主要研究者简历	
主审委员：郑雪咏、王一帆	
审查结果： ■同意 □修正后同意 □终止或暂停已批准的研究 □不同意	
审评意见：<br>同意进行该项临床研究。<br>主任委员：<br>日期：2021.6.25	
注意：<br>本伦理委员会是独立的，并遵循 ICH GCP 原则、中国 GCP 及当地相关法规。<br>凡涉及人类遗传资源采集、收集、买卖、出口、出境审批的项目需在我院人体生物标本及人类遗传资源管理委员会审批。<br>研究过程中若变更主要研究者，以及对临床研究方案、知情同意书、招募材料等修改，请提交修正案审查申请。<br>发生严重不良事件，请在获知后 24 小时内提交严重不良事件报告。<br>发生违背方案的情况，请及时提交违背方案报告。<br>本批件自签发之日起生效，请按照伦理委员会规定的跟踪审查频率，在截止日期前 1 个月提交研究进展报告。<br>申请人暂停或提前终止临床研究，请及时提交暂停/终止研究报告。<br>完成临床研究，请提交研究完成报告。	

地址：杭州市庆春东路 3 号 邮编：310016 电话：86 571 86006811 传真：86 571 86960497
